# Altered Clock and Lipid Metabolism-Related Genes in Atherosclerotic Mice Kept with Abnormal Lighting Condition

**DOI:** 10.1155/2016/5438589

**Published:** 2016-08-18

**Authors:** Zhu Zhu, Bingxuan Hua, Zhanxian Shang, Gongsheng Yuan, Lirong Xu, Ermin Li, Xiaobo Li, Ning Sun, Zuoqin Yan, Ruizhe Qian, Chao Lu

**Affiliations:** ^1^Department of Physiology and Pathophysiology, School of Basic Medical Sciences, Fudan University, Shanghai 200032, China; ^2^Department of Orthopedics, Zhongshan Hospital, Fudan University, Shanghai 200032, China

## Abstract

*Background*. The risk of atherosclerosis is elevated in abnormal lipid metabolism and circadian rhythm disorder. We investigated whether abnormal lighting condition would have influenced the circadian expression of clock genes and clock-controlled lipid metabolism-related genes in ApoE-KO mice.* Methods*. A mouse model of atherosclerosis with circadian clock genes expression disorder was established using ApoE-KO mice (ApoE-KO LD/DL mice) by altering exposure to light. C57 BL/6J mice (C57 mice) and ApoE-KO mice (ApoE-KO mice) exposed to normal day and night and normal diet served as control mice. According to zeitgeber time samples were acquired, to test atheromatous plaque formation, serum lipids levels and rhythmicity, clock genes, and lipid metabolism-related genes along with Sirtuin 1 (Sirt1) levels and rhythmicity.* Results*. Atherosclerosis plaques were formed in the aortic arch of ApoE-KO LD/DL mice. The serum lipids levels and oscillations in ApoE-KO LD/DL mice were altered, along with the levels and diurnal oscillations of circadian genes, lipid metabolism-associated genes, and Sirt1 compared with the control mice.* Conclusions*. Abnormal exposure to light aggravated plaque formation and exacerbated disorders of serum lipids and clock genes, lipid metabolism genes and Sirt1 levels, and circadian oscillation.

## 1. Introduction

All species on the earth exhibit circadian rhythms synchronized with the environmental cycles of day and night. In mammals, the circadian pattern consists of a central clock located in the suprachiasmatic nucleus (SCN) and a peripheral clock spread over almost all of the major organ systems including liver and fat [1, 2]. The SCN senses the external light signal and releases neurotransmitters and hormones in response, which regularly influence the circadian expression of the clock genes in the peripheral tissues [3]. These peripheral oscillators play a critical tissue-specific role [4]. The circadian rhythm of animals is manifested via feedback loop formed by a number of clock genes. The main mammalian circadian clock genes include circadian locomotor output cycles kaput* (Clock)*, brain and muscle encoding Arnt-like protein 1* (Bmal1)*, nuclear receptor subfamily 1, group D, member 1* (Rev-erb α)*, period* (Per1, Per2, Per3)*, and cryptochrome* (Cry1, Cry2)*. Many physiological activities are synchronized in an orderly manner by regulating the clock genes and their controlled genes. Hundreds of metabolism-related genes show circadian rhythms and most of them are controlled directly or indirectly by clock genes [5]. The peroxisome proliferator-activated receptors* (PPARs)*, RAR-related orphan receptor *α (ROR α),* and retinoid X receptor *α (RXR α)* are associated with lipid metabolism and exhibit circadian oscillations [6–9].

Currently, atherosclerotic cardiovascular disease (ASCVD) is a major health threat [10] and the primary contributor to death worldwide. The increasing burden of atherosclerotic cardiovascular risk is attributed to the escalating incidence of obesity, hypercholesterolemia, and dyslipidemia [11]. Cholesterol is a key component of arterial plaques [12] and liver is the major seat of cholesterol metabolism. Further, abdominal visceral fat has been strongly associated with cardiovascular risk [13, 14].

Studies demonstrate that ASCVD has a close relationship with circadian clocks. Disordered circadian rhythms induce vascular impairment and aggravate atherosclerosis [15]. Humans and mice with dysfunctional circadian oscillation are more likely to manifest cardiovascular diseases, while rats with hypertension also have irregular circadian gene expression [15–17]. Shift work accelerates atherosclerosis, which is a major manifestation of the pathophysiology underlying cardiovascular disease [18]. The deleterious effects of shift work on subclinical atherosclerosis are apparent in men before the age of 40 [19].

Sirtuin 1* (Sirt1)* a nuclear protein targeting histone H3 and H4 deacetylation [20] and its enzymatic activity also exhibit a circadian change [21]. Further,* Sirt1* plays a significant protective role in atherosclerosis [22].* Sirt1* especially regulates hepatic lipid metabolism by inhibiting fat synthesis and promoting fat oxidation [23–25]. It increases fat mobilization in white fat tissue by inhibiting* PPAR γ* [26].

The circadian clock disorder of atherosclerotic mice has been found in mouse SCN, heart, and liver [27]. It is unclear whether peripheral clocks, lipid metabolism-related genes, and atheroprotective factor* Sirt1* are altered in the liver and fat of atherosclerotic mice. We generated a mouse model of atherosclerotic via abnormal alteration in day and night and tested for atherosclerotic plaque formation and circadian expression in clock genes. We also examined the diurnal expression of clock genes* Bmal1*,* Per2*, and* Cry1 Rev-erbα*, as well as lipid metabolism-related* PPARα*,* PPARγ*,* RORα*,* RXR α*, and* Sirt1*.

## 2. Materials and Methods

### 2.1. Animal Model

We selected 10-week-old male C57BL/6J mice (Laboratory Animal Research Center of Chinese Academy of Sciences, Shanghai, China) and the age and gender were matched to ApoE-KO mice (Beijing Laboratory Animal Research Center, Beijing, China). ApoE-KO mouse is an ideal model of atherosclerosis [28, 29]. And the abnormal light stimulation leads to circadian disorder [30]. Therefore, the atherosclerosis model manifesting circadian clock genes expression disorder was built by altering exposure to environmental light. The ApoE-KO mice were randomly divided into two groups: (1) mice (ApoE-KO group) exposed to normal day-night pattern and (2) mice (ApoE-KO LD/DL group) maintained on a light-dark (LD) cycle (12 h light/12 h dark) for 2 weeks, followed by a dark-light (DL) cycle for 2 weeks, and then transferred to another LD cycle for 2 weeks. All mice were free to obtain regular diet and water. All the samples were obtained from mice at the end of the six weeks. According to external cues called zeitgeber times (ZT stands for standardized notation for the time during an entrained circadian cycle, ZT0 represents the start of the light phase, and ZT12 is the beginning of the dark phase, during a 24-hour light-dark cycle), we controlled light on at 08:00 which was designated as zeitgeber time 0 (ZT0) and light off at 20:00 (ZT12). The liver and fat tissues were obtained at different time points including ZT0, ZT4, ZT8, ZT12, ZT16, and ZT20 (four mice per time point). The samples were immediately frozen in liquid nitrogen and stored at −80°C. All the animal experiments were performed according to the criteria of the Medical Laboratory Animal Administrative Committee of Shanghai. This study was approved by the Medical Ethics Committee of Fudan University, Shanghai, China.

### 2.2. Analysis of Mouse Serum Lipids

The serum was used for lipid detection. The mice were generally anesthetized by administering chloral hydrate at ZT0, ZT4, ZT8, ZT12, ZT16, and ZT20. Blood was immediately collected after removing the whole eye ball. Subsequently, mice were euthanized with pentobarbital sodium through intraperitoneal injection. The serum was collected by centrifugation for 30 min at 4°C followed by serum lipid detection. The levels of glucose, triglyceride, total cholesterol, and low-density lipoprotein (LDL) in serum were measured by enzymatic methods [14]. The kits were purchased from Rongsheng Biotechnology Company Ltd. (Shanghai, China). We tested the samples according to the manufacturer's instruction.

### 2.3. Tissue Staining

To investigate the effect of abnormal light stimulation on atheromatous plaque formation in mice, we used Oil Red O staining to determine vulnerable plaque formation as previously described [30]. The arterial arcades were carefully dissected and fixed in 4% paraformaldehyde for 24 h at room temperature. The fixed samples were rinsed three times, each time for 15 minutes. The aortic segments were dehydrated in 20% sugar solution for 24 h and 30% sucrose solution for 24 h. Subsequently, the samples were embedded in Tissue-Tek OCT compound. Serial sections of 10 *μ*m cross-section were prepared and fixed at 4°C using 70% ethanol solution and staining for 1 min in Oil Red O (Sigma-Aldrich, O0625, USA) to identify atherosclerotic plaques. Oil Red O was dissolved in isopropanol and mixed in 3 : 2 (v/v) (water to Oil Red O) and the plaques were stained for 30 min. Then the samples were carefully washed for three times, each time for 5 minutes and stained for 3~5 min in hematoxylin (Beyotime, C0107, China).

### 2.4. Total RNA Extraction and Reverse Transcription

Total RNA from liver and fat were isolated with TRIzol reagent (Invitrogen Life Technologies), according to the manufacturer's instructions. The quantity of total RNA was determined using UV spectrophotometry. RNA integrity was assessed using agarose gel electrophoresis, followed by reverse transcription of 1 *μ*g of total RNA. The amplified first-strand cDNA was prepared with oligo-dT primers using a commercial cDNA synthesis kit (ReverTra Ace qPCR RT kit, Toyobo, Osaka, Japan). The cDNA was subsequently amplified for 35 cycles using specific primers (Table 1).

### 2.5. Real-Time PCR

The cDNA was diluted to 10 ng/*μ*L and PCR reactions were amplified on a real-time PCR machine (Bio-Rad, Hercules, CA, USA). The PCR reaction mixture included 2.5 *μ*L of 10 ng/*μ*L cDNA, 1 *μ*L of 10 *μ*M primers (0.5 *μ*L of upstream primers, 0.5 *μ*L of downstream primers), 10 *μ*L SYBR Green real-time PCR Master mix (Bio-Rad, Hercules, CA, USA), and 7.5 *μ*L of H_2_O, in a final volume of 20 *μ*L. The samples were subjected to 35 cycles of amplification. The amplification protocol included denaturation at 94°C for 15 sec, primer annealing for 30 sec, and extension at 72°C for 30 sec. Melting profiles were performed following each PCR reaction. The relative quantification of gene expression was analyzed from the measured threshold cycles (CT) using the 2^−ΔΔCT^ method. The CT value represents the number of cycles at the cross-point of ΔRn versus threshold. The higher the CT value, the greater the number of steady cycles and the lower the gene expression. As the amplification efficiencies of the target genes and the internal control were equal, the relative changes in the target gene expression of the altered clock genes in the mice compared with normal mice (ΔCT calibrator value) were calculated using the equation 2^−ΔΔCT^. The ΔCT values were determined by subtracting the average GAPDH CT value from the average target gene CT value.

### 2.6. Statistical Analysis

Each value represents mean ± SD. The values for mRNA levels are presented as relative values in all experiments. We employed single cosinor method to analyze circadian rhythm [17, 31], and the equation used for stating cosine function was shown as follows: *Y*(*t*) = *M* + *A∗*cos⁡(*x∗t* + *u*). The mesors, amplitudes, and acrophases were rhythm characteristics and estimated by this method. The mesor represents middle value of the fitted cosine. The amplitude is from half of the biggest differences (Value_max_ − Value_min_) in the fitted cosine function. And the acrophase means the peak value time. A probability value determined by one-way analysis of variance (ANOVA) of ≤ 0.05 indicated a statistically significant difference. Primer sequences of the target genes in the present study were found in GeneBank as shown in Table 1.

## 3. Results

### 3.1. Atheromatous Plaque Formation in the Arterial Arch

**Figure 1:**
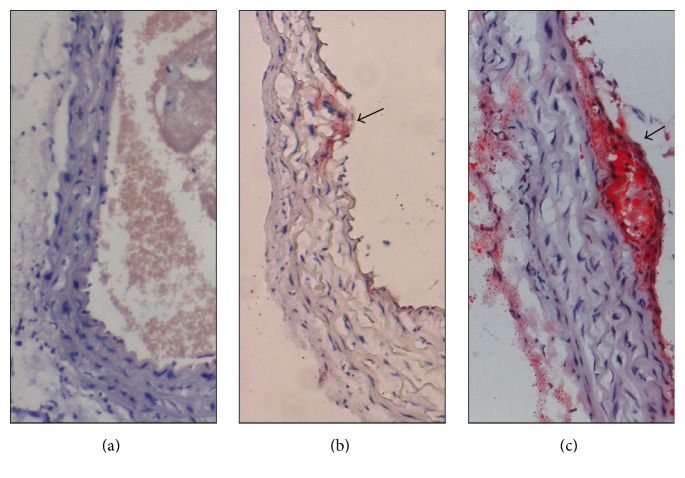
Oil Red O staining of the aortic arch of mice. We used Oil Red O staining to evaluate vulnerable plaques in the wild type C57 BL/6J (C57) mice, the ApoE knockout (ApoE-KO) mice treated with regular light-dark conditions, and the ApoE-KO (ApoE-KO LD/DL) mice with abnormal light stimulation. The arterial arch of C57, ApoE-KO, and ApoE-KO LD/DL mice was dyed with Oil Red O. No obvious changes were found in the C57 group (a). Endothelial lipid deposition and foam cell formation were seen in ApoE-KO group (b). However, exposure to abnormal day and night for 6 weeks resulted in lipid core and thicker caps were detected in ApoE-KO LD/DL mice (c). Arrowheads indicate lipid deposition. Original magnification ×200 (*n* = 4).

To investigate the effect of abnormal light stimulation on atheromatous plaque formation in mice, Oil Red O staining was used to determine vulnerable plaque formation. 10-week-old ApoE-KO mice were divided into two groups randomly. The wild type C57 BL/6J (C57) mice and one group of ApoE knockout (ApoE-KO) mice were exposed to regular light-dark conditions. The other group of ApoE-KO mice (ApoE-KO LD/DL) was exposed to abnormal light stimulation. Thus, three experimental groups were created. The arterial arch of C57 mice, ApoE-KO mice, and ApoE-KO LD/DL mice was dyed with Oil Red O (Figure 1). No pathological change was observed in C57 mice (Figure 1(a)). Compared with C57 mice, the ApoE-KO mice showed endothelial lipid deposition and foam cell formation (Figure 1(b)). In ApoE-KO LD/DL mice, lipid core and thicker cap were detected (Figure 1(c)).

### 3.2. Serum Lipid Level and Rhythm

We evaluated the serum lipid levels in mouse samples every 4 h in the C57, ApoE-KO, and ApoE-KO LD/DL mice at ZT0, ZT4, ZT8, ZT12, ZT16, and ZT20 (ZT0 corresponds to “lights on” and ZT12 to “light off”) (Table 2).

**Figure 2:**
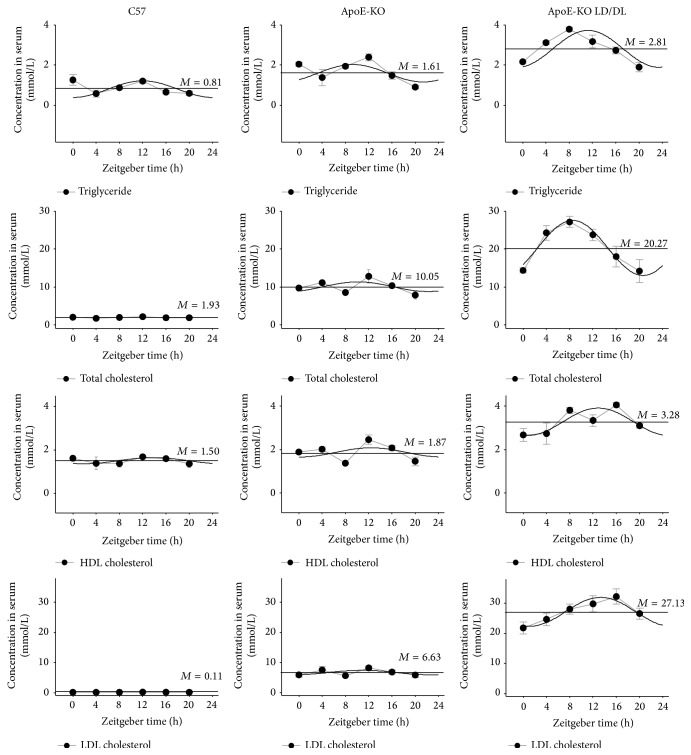
Serum total triglyceride, cholesterol, HDL-CHO, and LDL-CHO of blood in mice. We collected the blood of mice in three groups at ZT0, ZT4, ZT8, ZT12, ZT16, and ZT20. The total triglyceride (TG), total cholesterol (TC), and low-density lipoprotein (LDL) levels in ApoE-KO LD/DL mice were significantly increased compared with the C57 and ApoE-KO mice. Further, the mesors and amplitudes of TG, TC, HDL, and LDL were enhanced in ApoE-KO LD/DL mice compared with C57 mice and ApoE-KO mice (*n* = 4 for each group at every time point).

As shown in Figure 2, the total triglyceride (TG), total cholesterol (TC), and low-density lipoprotein (LDL) levels in ApoE-KO LD/DL mice were significantly increased compared with the C57 and ApoE-KO mice. Further, the mesors and amplitudes of TG, TC, HDL, and LDL were enhanced in ApoE-KO LD/DL mice compared with C57 mice and ApoE-KO mice. These results indicated that irregular light-dark interval aggravated the changes of lipid levels and oscillation patterns in ApoE-KO LD/DL mice.

### 3.3. Diurnal Expression Patterns of Circadian Genes in Liver and Fat

**Figure 3:**
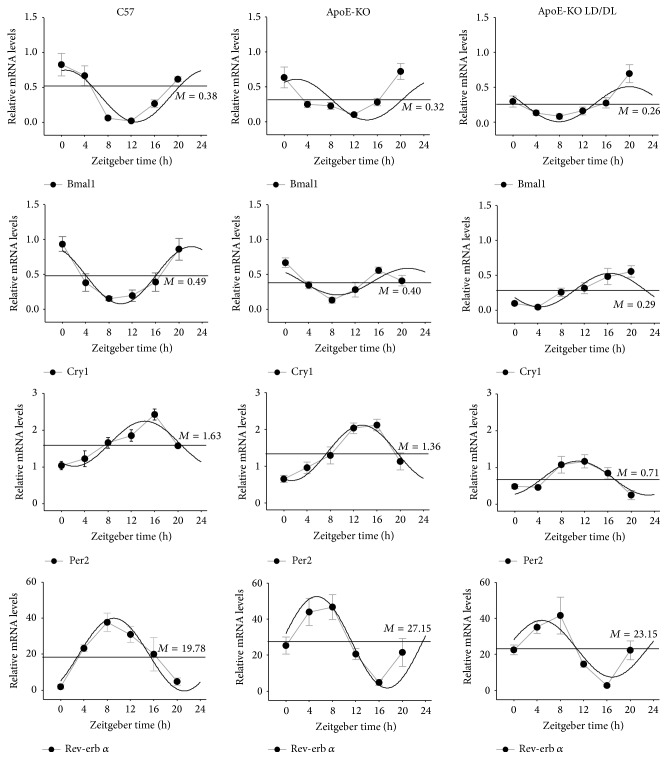
Circadian expression of clock genes in liver of C57, ApoE-KO, and ApoE-KO LD/DL mice. We obtained the liver tissues of mice at time points of ZT0, ZT4, ZT8, ZT12, ZT16, and ZT20. Levels of mRNA were determined by quantitative real-time PCR. In the liver tissue, the four clock genes* Bmal1*,* Per2*,* Cry1,* and* Rev-erb α* exhibit significant 24 h circadian expression pattern in C57, ApoE-KO, and ApoE-KO LD/DL mice. The mesors of* Bmal1* and* Per2* were reduced in ApoE-KO LD/DL mice compared with those in C57 and ApoE-KO mice. The amplitudes of Cry1 in ApoE-KO mice and ApoE-KO LD/DL mice were decreased compared with those in C57 mice, and the amplitudes of* Per2* and Rev-erb *α* in ApoE-KO LD/DL mice were attenuated compared with those in ApoE-KO mice. The acrophases of* Bmal1*,* Cry1,* and* Per2* in ApoE-KO LD/DL mice were altered compared with those in C57 and ApoE-KO mice. The mRNA levels of clock genes were normalized to* GAPDH* mRNA (*n* = 4 for each group at every time point).

**Figure 4:**
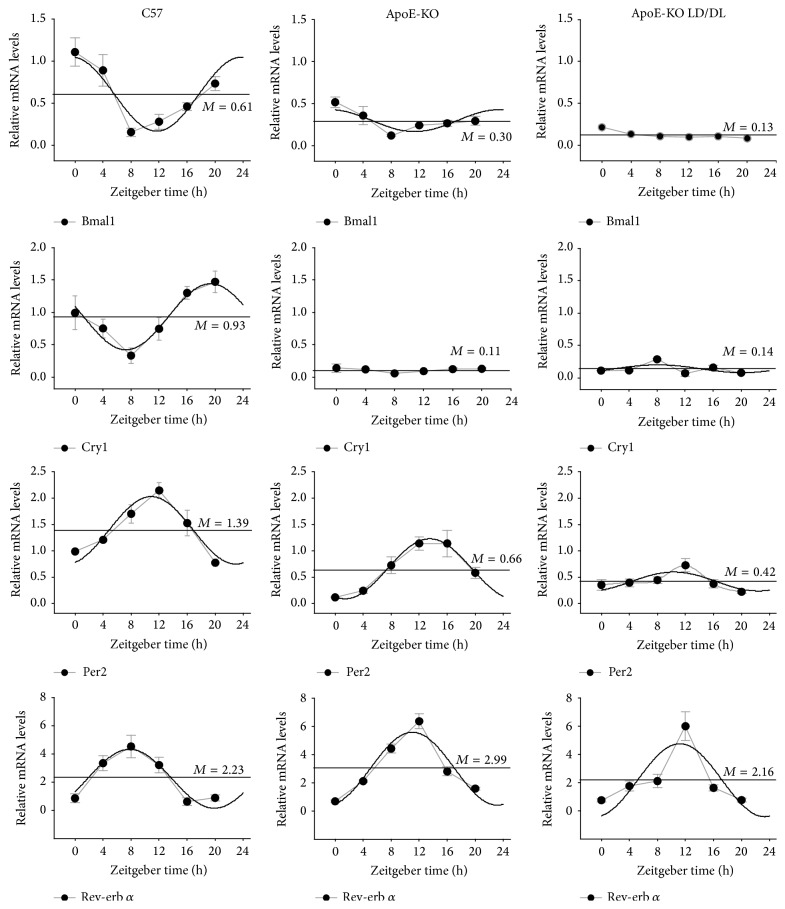
Circadian expression of clock genes in fat of C57, ApoE-KO, and ApoE-KO LD/DL mice. We obtained the epididymal fat tissues of mice at time points of ZT0, ZT4, ZT8, ZT12, ZT16, and ZT20. Levels of mRNA were determined by quantitative real-time PCR. In the epididymal fat tissue, circadian rhythm was lost in* Bmal1* of ApoE-KO LD/DL mice and* Cry1* of both ApoE-KO and ApoE-KO LD mice. The mesors were reduced of those four clock genes in ApoE-KO LD/DL mice compared with C57 mice. The amplitude of* Per2* in ApoE-KO LD/DL mice was significantly decreased compared with C57 and ApoE-KO LD/DL mice. The acrophases of* Rev*-*erbα* were delayed in ApoE-KO LD/DL mice compared with those in C57. The mRNA levels of clock genes were normalized to* GAPDH* mRNA (*n* = 4 for each group at every time point).

Liver and fat play an important role in energy metabolism. Abnormal energy metabolism increases the risk for atherosclerosis. Most of the clock genes exhibit circadian expression not only in SCN but also in peripheral tissues including liver and fat. To explore the possible changes in clock gene expression in atherosclerotic liver and fat, we performed real-time PCR analysis and compared the expression levels of the core clock genes in liver and fat, including* Bmal1*,* Cry1*,* Per2,* and* Rev-erbα*, in the three groups (C57, ApoE-KO, and ApoE-KO LD/DL) of mice at ZT0, ZT4, ZT8, ZT12, ZT16, and ZT20 as shown in Figures 3 and 4.

In the liver tissues, these four clock genes exhibited a significant 24 h circadian expression pattern (Figure 3). The mesors of* Bmal1* and* Per2* were reduced in ApoE-KO LD/DL mice compared with those in C57 and ApoE-KO mice. The amplitudes of* Cry1* in ApoE-KO mice and ApoE-KO LD/DL mice were decreased compared with those in C57 mice, and the amplitudes of* Per2* and* Rev-erb α* in ApoE-KO LD/DL mice were attenuated compared with those in ApoE-KO mice. The acrophases of* Bmal1*,* Cry1,* and* Per2* in ApoE-KO LD/DL mice were altered compared with those in C57 and ApoE-KO mice (Table 3).

In fat tissue, the circadian rhythmicity was lost in* Bmal1* of ApoE-KO LD/DL mice and in* Cry1* of ApoE-KO and ApoE-KO LD/DL mice (Figure 4). The mesors were reduced of those four clock genes in ApoE-KO LD/DL mice compared with C57 mice. The amplitude of* Per2* in ApoE-KO LD/DL mice was significantly decreased compared with C57 and ApoE-KO mice. Further, the acrophases of* Rev-erb α* were delayed in ApoE-KO LD/DL mice compared with those in C57 mice. Generally, the expression of* Bmal1 and Per2* in ApoE-KO LD/DL mice was significantly decreased compared with that in C57 mice and in ApoE-KO mice (Table 4). Overall, our data indicated that the abnormal day-night exposure aggravated the circadian genes expression disorder in liver and fat tissues of ApoE-KO LD/DL mice.

### 3.4. Diurnal Expression Pattern of PPAR *α*, PPAR *γ*, ROR *α*, and RXR *α* in Liver and Fat

Altered circadian gene expression contributed to changes in expression patterns of a series of downstream clock-controlled genes. Therefore, using quantitative RT-PCR, we examined the expression of* PPAR α*,* PPAR γ*,* ROR α,* and* RXR α* in liver and fat of atherosclerotic mice.

**Figure 5:**
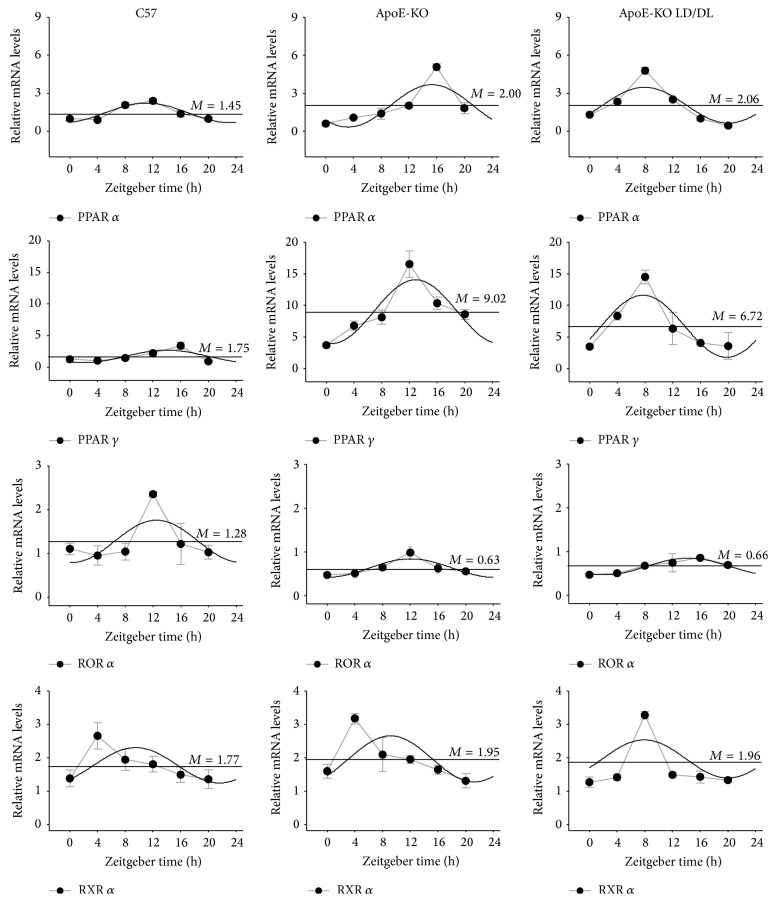
Circadian expression of* PPAR α*,* PPAR γ*,* ROR α,* and* RXR α* in mouse liver tissues. We also identified the diurnal expression of lipid metabolism-associated genes (*PPAR α*,* PPAR γ*,* ROR α,* and* RXR α*) in liver at specific time points. Levels of mRNA were determined by quantitative real-time PCR. The mesors of* PPAR α* and* PPAR γ* in ApoE-KO mice and ApoE-KO LD/DL mice were improved compared with those in C57 mice. The amplitudes of* PPAR α* and* PPAR γ* were enhanced and the amplitudes of* ROR α* were attenuated in ApoE-KO mice and ApoE-KO LD/DL mice compared with C57 mice. The acrophases of* PPAR α* and* PPAR γ* were advanced and the acrophase of* RXR α* was delayed in ApoE-KO LD/DL mice compared with C57 and ApoE-KO mice. The expression levels of* PPAR α* and* PPAR γ* in ApoE-KO LD/DL mice were increased compared with C57 mice. The mRNA levels of clock genes were normalized to* GAPDH* mRNA (*n* = 4 for each group at every time point).

As shown in Figure 5, in the liver tissue, the mesors of* PPAR α* and* PPAR γ* in ApoE-KO mice and ApoE-KO LD/DL mice were improved compared with those in C57 mice. The amplitudes of* PPAR α* and* PPAR γ* were enhanced while the amplitudes of* ROR α* were attenuated in ApoE-KO mice and ApoE-KO LD/DL mice compared with C57 mice. The acrophases of* PPAR α* and* PPAR γ* were advanced and the acrophase of* RXR α* was delayed in ApoE-KO LD/DL mice compared with C57 and ApoE-KO mice. The expression levels of* PPAR α* and* PPAR γ* in ApoE-KO LD/DL mice were increased compared with C57 mice (Table 3).

**Figure 6:**
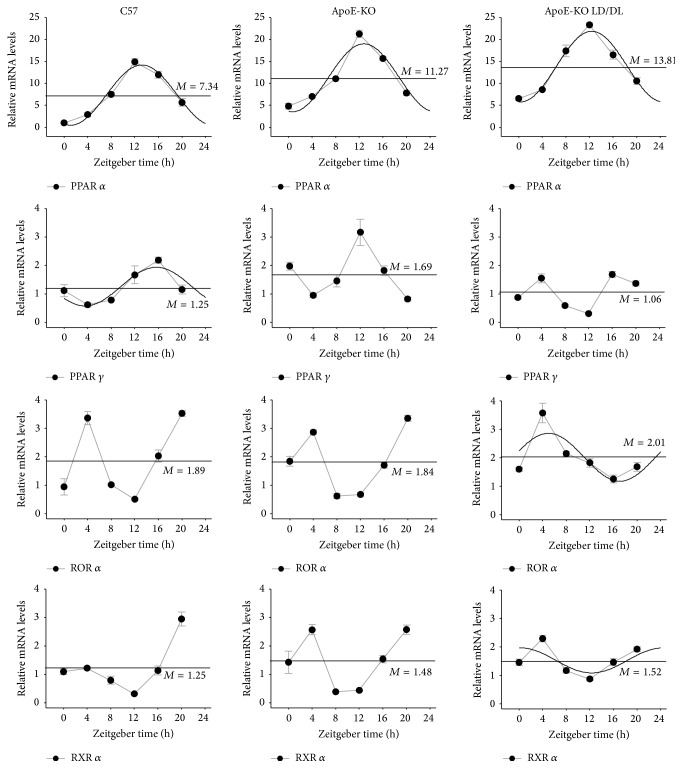
Circadian expression of* PPAR α*,* PPAR γ*,* ROR α,* and* RXR α* in mouse fat tissues. We also identified the diurnal expression of lipid metabolism-associated genes (*PPAR α*,* PPAR γ*,* ROR α,* and* RXR α*) in fat tissue at specific time points. Levels of mRNA were determined by quantitative real-time PCR. In the fat tissue,* PPAR γ* in ApoE-KO LD/DL mice and* RXR α* in C57 mice exhibited circadian oscillation not in accordance with 24 hours, and* PPAR γ* in ApoE-KO mice,* ROR α* in C57 and ApoE-KO mice, and* RXR α* in ApoE-KO mice did not well fit the cosine curves. The mesor of* PPARα* was enhanced in ApoE-KO LD/DL mice compared with those in C57 and ApoE-KO mice. The mRNA levels of clock genes were normalized to* GAPDH* mRNA (*n* = 4 for each group at every time point).

In the fat tissue,* PPAR γ* in ApoE-KO LD/DL mice and* RXR α* in C57 mice exhibited circadian oscillation not in accordance with 24 hours, and* PPAR γ* in ApoE-KO mice,* ROR α* in C57 and ApoE-KO mice, and* RXR α* in ApoE-KO mice did not well fit the cosine curves (Figure 6). The mesor of* PPARα* was enhanced in ApoE-KO LD/DL mice compared with those in C57 and ApoE-KO mice (Table 4). These results indicated that the oscillation of lipid metabolism-associated genes* PPAR α*,* PPAR γ*,* ROR α,* and* RXR α* was altered.

### 3.5. Diurnal Expression Pattern of Sirt1 in Liver and Fat

**Figure 7:**
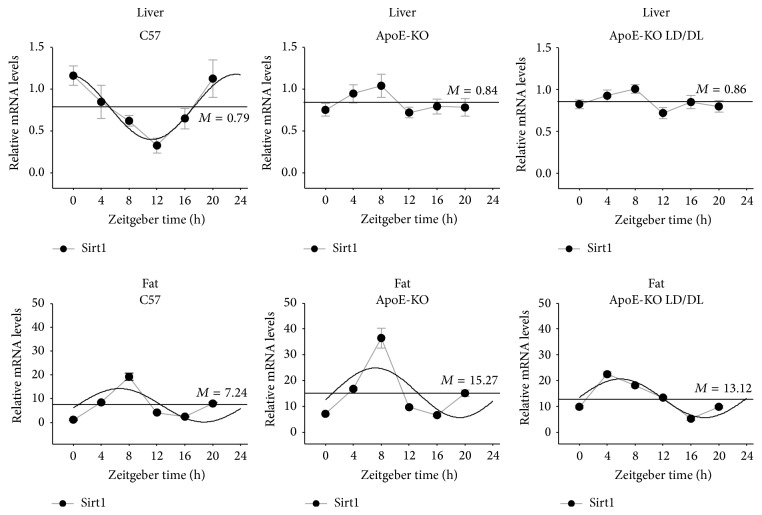
Circadian expression of* Sirt1* in liver and fat tissue of mice. Levels of* Sirt1* were determined by quantitative real-time PCR. In the liver tissue,* Sirt1* in ApoE-KO and ApoE-KO LD/DL mice exhibited circadian oscillation not in accordance with 24 hours. Further, in the fat tissue, the mesors of ApoE-KO mice and ApoE-KO LD/DL mice were increased compared with those in C57 mice. The expression of ZT0, ZT4, and ZT12 was promoted compared with C57 and ApoE-KO mice. The mRNA levels of clock genes were normalized to* GAPDH* mRNA (*n* = 4 for each group at every time point).

Sirtuin 1* (Sirt1)*, a nuclear protein, regulates liver and fat metabolism, and its HDAC activity also exhibits circadian oscillation in liver tissue [21]. We detected the* Sirt1* expression patterns in liver and fat as shown in Figure 7. In the liver tissue,* Sirt1* in ApoE-KO and ApoE-KO LD/DL mice exhibited circadian oscillation not in accordance with 24 hours. Further, in the fat tissue, the mesors of ApoE-KO mice and ApoE-KO LD/DL mice were increased compared with those in C57 mice. Concurrently, the expression of ZT0, ZT4, and ZT12 was promoted compared with C57 and ApoE-KO mice. Overall, the circadian oscillation of Sirt1 was lost in liver and altered in adipose tissues in ApoE-KO LD/DL mice.

## 4. Discussion

In this study, we investigated the role of abnormal day-night exposure in inducing atherosclerotic plaques in the aortic arch of ApoE-KO LD/DL mice. We also monitored the serum concentration and circadian changes in total triglyceride, total cholesterol, and low-density lipoprotein in ApoE-KO LD/DL mice compared with C57 mice and ApoE-KO mice. Further, the circadian oscillations of clock genes (*Bmal1*,* Per2*,* Cry1,* and* Rev-erbα*), lipid metabolism-associated genes (*PPAR α*,* PPAR γ*,* RXR α,* and* ROR α*), and* Sirt1* were modified extensively in ApoE-KO LD/DL mice compared with those in C57 mice and ApoE-KO mice.

Atherosclerosis is a chronic disease caused by multiple factors. The risk factors include hyperlipidemia, hypertension, smoking, diabetes, vascular inflammation, and autoimmune disease. High lipid levels, especially LDL, increase the risk of atherosclerosis [32, 33]. Therefore, we sought to identify the circadian alteration in clock genes and lipid metabolism-related genes altered in the liver and fat of atherosclerotic mice.

ApoE-KO mouse is an ideal model of atherosclerosis, in which total serum cholesterol and low-density lipoprotein levels are significantly higher than in C57 mice of the same sex. Further, ApoE-KO mice displayed significant atherosclerotic plaques in the aorta at six months [34]. Our preliminary studies also showed that, in 10-week-old male ApoE-KO mice fed with Western diet and exposed to chaotic light for six weeks, significant atherosclerotic plaques formed [30]. Further, the amplitudes of circadian oscillations in serum lipids were increased significantly with higher lipid levels. However, under similar conditions no obvious pathological changes in aorta were observed in C57 mice of the same sex and age [30]. All those results agreed with our research.

Atherosclerosis is an inflammatory disease involved in accumulating of lipid in the arterial wall.* PPAR α* and* PPAR γ* control almost all phases of atherosclerosis formation.* PPAR α* decreased the density of LDL in patients with dyslipidemia [35], and its activation repressed the accumulation of oxidized-LDL in atherosclerotic plaques of insulin-resistant mice [36]. In this study, the total expression level of* PPARα* was increased dramatically compared to both in liver and in fat tissue of C57 mice. In addition, the amplitudes of* PPARα* were advanced in liver of ApoE-KO LD/DL mice compared with the control groups.* PPAR γ* agonist inhibited phosphorylation of NF-*κ*B to repress the inflammatory response [37, 38]. In atherosclerotic mice, the amplitudes of* PPARγ* in liver were significantly changed compared with the control groups, and the acrophase was delayed compared with control mice.* ROR α* has been reported to play important roles in circadian system, adipocyte glyceroneogenesis, and liver gluconeogenesis [39]. It could inhibit NF-*κ*B activation to attenuate the inflammatory response, which might act as a potent object in the treatment of atherosclerosis [40]. And* ROR α* was positively correlated with HDL levels [41]. In our study the amplitudes of* ROR α* in liver were attenuated in ApoE-KO mice and ApoE-KO LD/DL mice compared with C57 mice. Diabetes promotes oxidative stress to aggravate atherosclerosis [42], and* RXR* agonists postponed the atherosclerosis progression [43]. In liver tissue, the acrophase of* RXR α* was delayed in ApoE-KO LD/DL mice compared with C57 and ApoE-KO mice. The results indicated that the expression patterns of* PPAR α*,* PPAR γ*,* ROR α,* and* RXR α* in mice were altered in ApoE-KO mice, which would promote atherosclerosis progression.

*Sirt1* plays a critical role in circadian rhythm and in atherosclerosis. In liver tissue, the rhythms of ApoE-KO and ApoE-KO LD/DL mice were lost. And the mesors of* Sirt1* were changed in ApoE-KO LD/DL mice compared with C57.* Sirt1* may serve as a link between the clock genes controlling circadian alteration and lipid-related gene oscillation, and its expression disorder might aggravate atherosclerosis. On the other hand, shifted food intake time induced by light at night could eventually lead to obesity in mice [44] and the timing of food intake affected the rhythm of clock genes in liver and other peripheral organs [45]. The timing of feed might be the mechanism of light shifts inducing circadian genes expression disorder and contributing to atherosclerosis.

## 5. Conclusion

We detected altered expression of the circadian clock genes and lipid metabolism-related genes in liver and fat tissues of atherosclerotic mice manifesting circadian genes expression disorder for the first time. We also observed normal circadian rhythm in* Sirt1* expression and its changes in liver and fat tissues of atherosclerotic mice with disordered circadian genes expression. Specific mechanisms underlying the phenomenon remain to be further studied. Our findings provide new research directions and lay a theoretical foundation for further study of the relationship between circadian clock, lipid metabolism disorders, and atherosclerosis.

## Figures and Tables

**Table 1:** Primer pairs used to amplify PCR products.

Gene name	Annealing temperature	GeneBank accession number	Forward primer (5′-3′)
Bmal1	60°C	NM_007489	Forward: CACTGACTACCAAGAAAGTATG Reverse: ATCCATCTGCTGCCCTGAGA
Per2	58°C	NM_011066	Forward: CAGACTCATGATGACAGAGG Reverse: GAGATGTACAGGATCTTCCC
Rev-erb *α*	60°C	NM_145434	Forward: TACATTGGCTCTAGTGGCTCC Reverse: CAGTAGGTGATGGTGGGAAGTA
Cry1	58°C	NM_007771	Forward: CACTGGTTCCGAAAGGGACTC Reverse: CTGAAGCAAAAATCGCCACCT
PPAR *α*	61°C	NM_011144	Forward: TCGGCGAACTATTCGGCTG Reverse: GCACTTGTGAAAACGGCAGT
ROR *α*	61°C	NM_013646	Forward: GGGGACAATTTCTACTTCACTGG Reverse: GCAAACGGTAGTAAGGGCTG
GAPDH	60°C	BC_083149	Forward: ACAGCCGCATCTTCTTGTGCAGT Reverse: GGCCTTGACTGTGCCGTGAATTT

**Table 2:** Circadian rhythmic parameters of triglyceride, total cholesterol, HDL cholesterol, and LDL cholesterol levels in serum.

Serum lipid	Mesor	Amplitude	Acrophase ZT (h)
*C57 ND*			
Triglyceride	0.81 ± 0.12	0.10 ± 0.04	11.95 ± 0.90
Total cholesterol	1.93 ± 0.12	0.12 ± 0.06	13.37 ± 0.72
HDL cholesterol	1.50 ± 0.18	0.14 ± 0.04	13.75 ± 0.07
LDL cholesterol	0.11 ± 0.02	0.04 ± 0.01	13.33 ± 0.13

*ApoE-KO*			
Triglyceride	1.61 ± 0.16^*∗∗*^	0.43 ± 0.06^*∗∗*^	8.17 ± 0.17
Total cholesterol	10.05 ± 0.81^*∗∗*^	1.27 ± 0.31^*∗*^	10.12 ± 0.25^*∗*^
HDL cholesterol	1.87 ± 0.11^*∗*^	0.21 ± 0.04	12.34 ± 0.27
LDL cholesterol	6.63 ± 0.71^*∗∗*^	0.73 ± 0.27^*∗*^	10.81 ± 0.13^*∗∗*^

*ApoE-KO LD/DL*			
Triglyceride	2.81 ± 0.16^*∗∗*##^	0.91 ± 0.02^*∗∗*##^	11.15 ± 0.11
Total cholesterol	20.27 ± 1.82^*∗∗*##^	7.26 ± 0.42^*∗∗*##^	8.48 ± 0.08^*∗∗*^
HDL cholesterol	3.28 ± 0.22^*∗∗*##^	0.63 ± 0.13^*∗∗*##^	12.80 ± 0.15
LDL cholesterol	27.13 ± 2.08^*∗∗*##^	4.79 ± 0.49^*∗∗*##^	13.21 ± 0.05^##^

*n* = 4. ^*∗*^ *P* < 0.05, ^*∗∗*^ *P* < 0.01 versus C57 mice; ^##^ *P* < 0.01 versus ApoE-KO mice.

**Table 3:** Circadian rhythmic parameters of clock genes, PPAR *α*, PPAR *γ*, ROR *α*, RXR *α*, and Sirt1 in mouse liver.

Gene	Mesor	Amplitude	Acrophase ZT (h)
*C57 ND*			
Bmal1	0.38 ± 0.02	0.37 ± 0.05	0.69 ± 0.06
Cry1	0.49 ± 0.03	0.41 ± 0.04	21.98 ± 0.15
Per2	1.63 ± 0.08	0.61 ± 0.20	14.20 ± 0.25
Rev-erb *α*	19.78 ± 5.65	20.12 ± 7.8	9.09 ± 0.49
PPAR *α*	1.45 ± 0.09	0.77 ± 0.06	11.00 ± 0.07
PPAR *γ*	1.75 ± 0.36	0.97 ± 0.36	13.90 ± 0.17
ROR *α*	1.28 ± 0.10	0.48 ± 0.04	12.42 ± 0.12
RXR *α*	1.77 ± 0.15	0.53 ± 0.05	6.38 ± 1.42
Sirt1	0.79 ± 0.04	0.39 ± 0.05	23.16 ± 0.06

*ApoE-KO*			
Bmal1	0.32 ± 0.03	0.29 ± 0.06	0.38 ± 0.04
Cry1	0.40 ± 0.07	0.19 ± 0.02^*∗∗*^	21.32 ± 0.06
Per2	1.36 ± 0.13^*∗∗*^	0.75 ± 0.12	13.31 ± 0.10
Rev-erb *α*	27.15 ± 3.92^*∗*^	25.33 ± 6.63	4.98 ± 0.10^*∗∗*^
PPAR *α*	2.00 ± 0.16^*∗∗*^	1.67 ± 0.10^*∗∗*^	15.20 ± 0.06^*∗∗*^
PPAR *γ*	9.02 ± 0.36^*∗∗*^	5.01 ± 0.66^*∗∗*^	12.80 ± 0.05
ROR *α*	0.63 ± 0.05^*∗∗*^	0.21 ± 0.03^*∗∗*^	11.99 ± 0.11
RXR *α*	1.95 ± 0.11	0.69 ± 0.23	7.22 ± 0.08
Sirt1	—	—	—

*ApoE-KO LD/DL*			
Bmal1	0.26 ± 0.04^*∗∗*##^	0.25 ± 0.09	19.87 ± 0.03^*∗∗*##^
Cry1	0.29 ± 0.02^*∗∗*^	0.24 ± 0.04^*∗∗*^	16.27 ± 0.10^*∗∗*##^
Per2	0.71 ± 0.06^*∗∗*##^	0.46 ± 0.08^#^	10.77 ± 0.08^*∗*##^
Rev-erb *α*	23.15 ± 2.42	15.7 ± 2.30^#^	4.78 ± 0.07^*∗∗*^
PPAR *α*	2.06 ± 0.06^*∗∗*^	1.61 ± 0.02^*∗∗*^	7.87 ± 0.02^*∗∗*##^
PPAR *γ*	6.72 ± 0.10^*∗∗*##^	4.89 ± 0.07^*∗∗*^	7.64 ± 0.02^*∗∗*##^
ROR *α*	0.66 ± 0.02^*∗∗*^	0.19 ± 0.02^*∗∗*^	14.17 ± 0.02^*∗*##^
RXR *α*	1.96 ± 0.05	0.44 ± 0.01	9.13 ± 0.05^*∗∗*#^
Sirt1	—	—	—

*n* = 4. ^*∗*^ *P* < 0.05, ^*∗∗*^ *P* < 0.01 versus C57 mice; ^#^ *P* < 0.05, ^##^ *P* < 0.01 versus ApoE-KO mice.

**Table 4:** Circadian rhythmic parameters of clock genes, PPAR *α*, PPAR *γ*, ROR *α*, RXR *α*, and Sirt1 in mouse fat.

Gene	Mesor	Amplitude	Acrophase ZT (h)
*C57 ND*			
Bmal1	0.61 ± 0.04	0.44 ± 0.05	23.58 ± 0.09
Cry1	0.93 ± 0.01	0.51 ± 0.04	19.22 ± 0.12
Per2	1.39 ± 0.08	0.64 ± 0.08	10.77 ± 0.08
Rev-erb *α*	2.23 ± 0.14	2.08 ± 0.19	7.75 ± 0.01
PPAR *α*	7.34 ± 0.16	6.83 ± 0.29	12.1 ± 0.03
PPAR *γ*	1.25 ± 0.05	0.68 ± 0.05	15.55 ± 0.03
ROR *α*	—	—	—
RXR X	—	—	—
Sirt1	7.24 ± 0.24	7.18 ± 0.28	6.33 ± 0.01

*ApoE-KO*			
Bmal1	0.30 ± 0.03^*∗∗*^	0.13 ± 0.03^*∗∗*^	23.27 ± 0.15
Cry1	—	—	—
Per2	0.66 ± 0.01^*∗∗*^	0.57 ± 0.09	13.41 ± 0.06^*∗∗*^
Rev-erb *α*	2.99 ± 0.08^*∗∗*^	2.58 ± 0.22^*∗*^	10.96 ± 0.02^*∗∗*^
PPAR *α*	11.27 ± 0.20^*∗∗*^	7.71 ± 0.10^*∗∗*^	12.68 ± 0.01
PPAR *γ*	—	—	—
ROR *α*	—	—	—
RXR *α*	—	—	—
Sirt1	15.27 ± 0.73^*∗∗*^	9.60 ± 0.97^*∗∗*^	6.10 ± 0.02

*ApoE-KO LD/DL*			
Bmal1	—	—	—
Cry1	—	—	—
Per2	0.42 ± 0.05^*∗∗*##^	0.18 ± 0.03^*∗∗*##^	10.28 ± 0.09^##^
Rev-erb *α*	2.16 ± 0.18^*∗*##^	2.01 ± 0.36^#^	11.12 ± 0.03^*∗∗*^
PPAR *α*	13.81 ± 0.33^*∗∗*##^	6.70 ± 0.05	12.03 ± 0.02
PPAR *γ*	—	—	—
ROR *α*	2.01 ± 0.04	0.84 ± 0.05	4.79 ± 0.02
RXR *α*	1.52 ± 0.04	0.44 ± 0.02	2.29 ± 0.07
Sirt1	13.12 ± 0.10^*∗∗*#^	7.47 ± 0.18	5.84 ± 0.01

*n* = 4. ^*∗*^ *P* < 0.05, ^*∗∗*^ *P* < 0.01 versus C57 mice; ^#^ *P* < 0.05, ^##^ *P* < 0.01 versus ApoE-KO mice.

## Abbreviations

Bmal1: Muscle Arnt-like protein 1
Per2: Period 2
Cry1: Cryptochrome 1
Rev-erb *α*: Nuclear receptor subfamily 1, group D, member 1
Clock: Circadian locomotor output cycles kaput
SCN: Suprachiasmatic nucleus
ASCVD: Atherosclerotic cardiovascular disease
PPAR *α*: Proliferator-activated receptors *α*
PPAR *γ*: Proliferator-activated receptors *γ*
ROR *α*: RAR-related orphan receptor *α*
RXR *α*: Retinoid X receptor *α*
Sirt1: Sirtuin 1
qRT-PCR: Quantitative real-time polymerase chain reaction
CCG: Clock-controlled genes
ZT: Zeitgeber time
SREBP-1c: Sterol-regulatory element binding protein 1 c
GAPDH: Glyceraldehyde-3-phosphate dehydrogenase.

## References

[1] Ralph M. R., Foster R. G., Davis F. C., Menaker M. (1990). Transplanted suprachiasmatic nucleus determines circadian period. *Science*.

[2] Bass J., Takahashi J. S. (2010). Circadian integration of metabolism and energetics. *Science*.

[3] Schibler U., Ripperger J., Brown S. A. (2003). Peripheral circadian oscillators in mammals: time and food. *Journal of Biological Rhythms*.

[4] Mendoza J., Pévet P., Challet E. (2008). High-fat feeding alters the clock synchronization to light. *Journal of Physiology*.

[5] Storch K.-F., Lipan O., Leykin I. (2002). Extensive and divergent circadian gene expression in liver and heart. *Nature*.

[6] Flavell D. M., Jamshidi Y., Hawe E. (2002). Peroxisome proliferator-activated receptor *α* gene variants influence progression of coronary atherosclerosis and risk of coronary artery disease. *Circulation*.

[7] Froy O. (2010). Metabolism and circadian rhythms-implications for obesity. *Endocrine Reviews*.

[8] Takahashi S., Inoue I., Nakajima Y. (2010). A promoter in the novel exon of hPPAR*γ* directs the circadian expression of PPAR*γ*. *Journal of Atherosclerosis and Thrombosis*.

[9] Beaven S. W., Tontonoz P. (2006). Nuclear receptors in lipid metabolism: targeting the heart of dyslipidemia. *Annual Review of Medicine*.

[10] Mozaffarian D., Benjamin E. J., Go A. S. (2015). Heart disease and stroke statistics—2015 update: a report from the American Heart Association. *Circulation*.

[11] Shay C. M., Ning H., Daniels S. R., Rooks C. R., Gidding S. S., Lloyd-Jones D. M. (2013). Status of cardiovascular health in US adolescents: prevalence estimates from the National Health and Nutrition Examination Surveys (NHANES) 2005–2010. *Circulation*.

[12] Linton M. R. F., Yancey P. G., Davies S. S., De Groot L. J., Beck-Peccoz P., Chrousos G. (2000). The role of lipids and lipoproteins in atherosclerosis. *Endotext*.

[13] Despres J.-P., Lemieux S., Lamarche B. (1995). The insulin resistance dyslipidemic syndrome: contribution of visceral obesity and therapeutic implications. *International Journal of Obesity*.

[14] Fox C. S., Massaro J. M., Hoffmann U. (2007). Abdominal visceral and subcutaneous adipose tissue compartments: association with metabolic risk factors in the framingham heart study. *Circulation*.

[15] Anea C. B., Zhang M., Stepp D. W. (2009). Vascular disease in mice with a dysfunctional circadian clock. *Circulation*.

[16] Ha M., Park J. (2005). Shiftwork and metabolic risk factors of cardiovascular disease. *Journal of Occupational Health*.

[17] Mohri T., Emoto N., Nonaka H. (2003). Alterations of circadian expressions of clock genes in Dahl salt-sensitive rats fed a high-salt diet. *Hypertension*.

[18] Xu J., Yang W., Deng Q., Huang Q., Yang J., Huang F. (2012). Flaxseed oil and *α*-lipoic acid combination reduces atherosclerosis risk factors in rats fed a high-fat diet. *Lipids in Health and Disease*.

[19] Puttonen S., Kivimäki M., Elovainio M. (2009). Shift work in young adults and carotid artery intima-media thickness: the cardiovascular risk in young Finns study. *Atherosclerosis*.

[20] Imai S.-I., Armstrong C. M., Kaeberlein M., Guarente L. (2000). Transcriptional silencing and longevity protein Sir2 is an NAD-dependent histone deacetylase. *Nature*.

[21] Nakahata Y., Kaluzova M., Grimaldi B. (2008). The NAD+-dependent deacetylase SIRT1 modulates clock-mediated chromatin remodeling and circadian control. *Cell*.

[22] Stein S., Matter C. M. (2011). Protective roles of SIRT1 in atherosclerosis. *Cell Cycle*.

[23] Baur J. A., Pearson K. J., Price N. L. (2006). Resveratrol improves health and survival of mice on a high-calorie diet. *Nature*.

[24] Lagouge M., Argmann C., Gerhart-Hines Z. (2006). Resveratrol improves mitochondrial function and protects against metabolic disease by activating SIRT1 and PGC-1*α*. *Cell*.

[25] Feige J. N., Lagouge M., Canto C. (2008). Specific SIRT1 activation mimics low energy levels and protects against diet-induced metabolic disorders by enhancing fat oxidation. *Cell Metabolism*.

[26] Picard F., Kurtev M., Chung N. (2004). Sirt1 promotes fat mobilization in white adipocytes by repressing PPAR-*γ*. *Nature*.

[27] Xu C., Lu C., Hua L. (2009). Rhythm changes of clock genes, apoptosis-related genes and atherosclerosis-related genes in apolipoprotein E knockout mice. *Canadian Journal of Cardiology*.

[28] Zhang S. H., Reddick R. L., Piedrahita J. A., Maeda N. (1992). Spontaneous hypercholesterolemia and arterial lesions in mice lacking apolipoprotein E. *Science*.

[29] Plump A. S., Smith J. D., Hayek T. (1992). Severe hypercholesterolemia and atherosclerosis in apolipoprotein E-deficient mice created by homologous recombination in ES cells. *Cell*.

[30] Hou L., Lu C., Huang Y., Chen S., Hua L., Qian R. (2009). Effect of hyperlipidemia on the expression of circadian genes in apolipoprotein E knock-out atherosclerotic mice. *Lipids in Health and Disease*.

[31] Nelson W., Tong Y. L., Lee J. K., Halberg F. (1979). Methods for cosinor-rhythmometry. *Chronobiologia*.

[32] Esterbauer H., Wag G., Puhl H. (1993). Lipid peroxidation and its role in atherosclerosis. *British Medical Bulletin*.

[33] McCrindle B. W., Urbina E. M., Dennison B. A. (2007). Drug therapy of high-risk lipid abnormalities in children and adolescents: a scientific statement from the American Heart Association atherosclerosis, hypertension, and obesity in youth committee, council of cardiovascular disease in the young, with the council on cardiovascular nursing. *Circulation*.

[34] Preitner N., Damiola F., Luis-Lopez-Molina (2002). The orphan nuclear receptor REV-ERB*α* controls circadian transcription within the positive limb of the mammalian circadian oscillator. *Cell*.

[35] Lemieux I., Laperrière L., Dzavik V., Tremblay G., Bourgeois J., Després J.-P. (2002). A 16-week fenofibrate treatment increases LDL particle size in type IIA dyslipidemic patients. *Atherosclerosis*.

[36] Verreth W., De Keyzer D., Pelat M. (2004). Weight loss-associated induction of peroxisome proliferator-activated receptor-*α* and peroxisome proliferator-activated receptor-*γ* correlate with reduced atherosclerosis and improved cardiovascular function in obese insulin-resistant mice. *Circulation*.

[37] Sasaki M., Jordan P., Welbourne T. (2005). Troglitazone, a PPAR-*γ* activator prevents endothelial cell adhesion molecule expression and lymphocyte adhesion mediated by TNF-*α*. *BMC Physiology*.

[38] De Bosscher K., Vanden Berghe W., Haegeman G. (2006). Cross-talk between nuclear receptors and nuclear factor *κ*B. *Oncogene*.

[39] Kadiri S., Monnier C., Ganbold M., Ledent T., Capeau J., Antoine B. (2015). The nuclear retinoid-related orphan receptor-*α* regulates adipose tissue glyceroneogenesis in addition to hepatic gluconeogenesis. *American Journal of Physiology—Endocrinology and Metabolism*.

[40] Delerive P., Monté D., Dubois G. (2001). The orphan nuclear receptor ROR*α* is a negative regulator of the inflammatory response. *EMBO Reports*.

[41] Vieira E., Ruano E. G., Figueroa A. L. C. (2014). Altered clock gene expression in obese visceral adipose tissue is associated with metabolic syndrome. *PLoS ONE*.

[42] Aoki M., Nata T., Morishita R. (2001). Endothelial apoptosis induced by oxidative stress through activation of NF-*κ*B: antiapoptotic effect of antioxidant agents on endothelial cells. *Hypertension*.

[43] Lin L., Peng F., Liu Y. (2016). Coadministration of VDR and RXR agonists synergistically alleviates atherosclerosis through inhibition of oxidative stress: An In Vivo and In Vitro Study. *Atherosclerosis*.

[44] Fonken L. K., Workman J. L., Walton J. C. (2010). Light at night increases body mass by shifting the time of food intake. *Proceedings of the National Academy of Sciences of the United States of America*.

[45] Dibner C., Schibler U., Albrecht U. (2009). The mammalian circadian timing system: organization and coordination of central and peripheral clocks. *Annual Review of Physiology*.

